# Development of microsatellite markers for the Japanese endemic conifer *Thuja standishii* and transfer to other East Asian species

**DOI:** 10.1186/s13104-019-4716-z

**Published:** 2019-10-25

**Authors:** James R. P. Worth, K. S. Chang, Y.-H. Ha, Aili Qin

**Affiliations:** 1 Department of Forest Molecular Genetics and Biotechnology, Forestry and Forest Products Research Institute, Matsunosato 1, Tsukuba, Ibaraki 305-8687 Japan; 2 Division of Forest Biodiversity and Herbarium, Korea National Arboretum, Pocheon, South Korea; 3 Institute of Forest Ecology, Environment and Protection, Chinese Academy of Forestry, Key Laboratory on Forest Ecology and Environmental Sciences of State Forestry and Grassland Administration, Beijing, 100091 China

**Keywords:** China, Conifer, Japanese arbor-vitae, Korea, Nuclear microsatellites, *Thuja*

## Abstract

**Objective:**

Design polymorphic microsatellite loci that will be useful for studies of the genetic diversity, gene-flow and reproduction in the Japanese endemic conifer *Thuja standishii* and test the transferability of these loci to the two other East Asian species, *T. sutchuenensis* and *T. koraiensis*.

**Results:**

Fifteen loci were developed which displayed 3 to 21 alleles per locus (average = 9.2) among 97 samples from three populations of *T. standishii*. Observed heterozygosity for all samples varied between 0.33 and 0.75 (average = 0.54) while expected heterozygosity values were higher with an average over the 15 loci of 0.62 (0.37–0.91). Low multi-locus probability of identity values (< 0.00002) indicate that these markers will be effective for identifying individuals derived from clonal reproduction. All 15 loci amplified in 13 samples of *T. sutchuenensis*, the sister species of *T. standishii*, with 1 to 11 alleles per locus (average = 4.33) while 13 loci amplified in four samples of the more distantly related *T. koraiensis* with 1 to 5 alleles per locus (average = 2.15).

## Introduction

*Thuja* is a small Cupressaceae genus consisting of five extant species with three species in East Asia and two in North America [6]. *Thuja standishii* (Gordon) Carriere (kurobe or nezuko in Japanese and Japanese arbor-vitae in English) is endemic to Japan where it has a scattered distribution from 40.67˚N to 33.49˚N on the islands of Honshu, Shikoku and Dogo (Shimane Prefecture). The species is most commonly a component of subalpine forests but also occurs in a variety of habitats including warm temperate forest (rarely), cool temperate forest, moorland and near the alpine zone. The species can grow as a single-stemmed tree up to 35 m tall but occurs as a multi-stemmed shrub under 1 m tall at the maximum limit of its elevational range [15]. It can purportedly reach a great age with trunks up to 3.5 m in diameter (Giant Tree Database, Biodiversity Center of Japan (http://kyoju.biodic.go.jp)) and individuals over 1000 years old.

Unlike most other Cupressaceae conifers of Japan, *T. standishii* has received little research attention with basic information on its conservation (including the impact of past logging), reproductive biology and genetic diversity, lacking. The rarity of forest dominated by *T. standishii* and its current insignificant role in forestry probably underlies this lack of research. However, the species is undoubtedly an important part of Japan’s biodiversity and cultural heritage. For example, it is one of Japan’s five precious timber trees (Kiso go-boku) that from the early 18th century were strictly protected from cutting in the Kiso region of central Honshu [8] and, in some parts of Japan, forests containing *T. standishii* are considered to represent the most untouched forests remaining in the landscape (e.g. [13]. Small population size and geographic isolation, its vulnerability to ring barking by deer and the impacts of past logging [15] has resulted in some populations being of conservation concern, especially in western Japan where the species is very rare [15]. One key aspect of the species biology that is poorly understood is the role of asexual reproduction in its regeneration. However, similarly to two other Japanese Cupressaceae conifers, *Thujopsis dolobrata* and *C. pisifera* that have been proven to regenerate clonally [3, 4], *T. standishii* also forms dense understory banks of juveniles (Worth personal observation) which may be clonally derived.

In the current age of genomics, microsatellites retain a vital role in biology due to their high information content, utility in a wide range of genetic applications and cost-efficient nature [5]. This study describes the development of Expressed Sequence Tagged (EST) nuclear microsatellite markers for *T. standishii* using next generation sequencing. These markers will be useful molecular tools to inform the conservation of this species via studies of its range-wide genetic diversity, gene flow and reproductive biology. In addition, the transferability of the markers was tested in the two other East Asian species for which no microsatellite markers have yet been developed including the Chinese endemic *Thuja sutchuenensis*, the sister species of *T. standishii* [7, 10], and *T. koraiensis*.

## Main text

### Materials and methods

Total RNA was extracted from an individual of *T. standishii* collected from the Forestry and Forest Products Research Institute Arboretum using a plant RNA isolation mini kit (Agilent Technologies, USA). An RNA-seq data set was constructed by the Beijing Genomics Institute on an Illumina HiSeq4000 platform. The *T. standishii* RNA-seq data consisted of 38,076,160 paired-end reads of 100 bp length. De novo assembly was undertaken in CLC Genomics Workbench 8.5.1 and the 53,614 resultant contigs (*N*50 = 1503 bp) were mined for microsatellite regions. Primers were developed bordering these regions with default settings using PrimerPro (http://webdocs.cs.ualberta.ca/∼yifeng/primerpro/). Microsatellites were selected if the number of tandem repeat units was greater than eight and if the microsatellite was located less than 25 bp from the beginning or end of the contig. These criteria resulted in 64 microsatellite primer pairs which were trialled for amplification in four samples. A total of 36 primer pairs successfully amplified and were subsequently tested for size heterogeneity in eight samples representative of the species range. For all loci, the forward primer was synthesized with one of three different M13 sequences (5′-GCCTCCCTCGCGCCA-3′, 5′-GCCTTGCCAGCCCGC-3′, and 5′-CAGGACCAGGCTACCGTG-3′), and the reverse was tagged with a pig-tail (5′-GTTTCTT-3′; [2]). The PCR reactions were performed following the standard protocol of the Qiagen Multiplex PCR Kit (Qiagen, Hilden, Germany), and consisted of a 10 uL reaction volume, containing approximately 5 ng of DNA, 5 uL of 2× Multiplex PCR Master Mix, and 0.06 uM of forward primer, 0.1 uM of reverse primer, and 0.08 uM of fluorescently labelled M13 primer. The PCR thermocycle consisted of an initial denaturation at 95 °C for 3 min; followed by 35 cycles of 95 °C for 30 s, 60 °C for 3 min, 68 °C for 1 min; and a 20 min extension at 68 °C. The PCR products were separated by capillary electrophoresis on an ABI3130 Genetic Analyzer (Life Technologies, Waltham, MA, USA) with the GeneScan 600 LIZ Size Standard (Life Technologies) and genotyping was done in GeneMarker (SoftGenetics, LLC, PA, USA). Overall, 15 loci were found to amplify reliably, display polymorphism and were readily scorable. The genetic variability of these 15 markers were tested in three populations from Atebi Daira Small Bird Forest, Mt Chausu Nature Park, in Nagano Prefecture (35.2286°N, 137.6673°E), Mt Torigata in Kouchi Prefecture (33.4936° N, 133.0638° E) and Mt Yamizo in Fukushima Prefecture (36.9343°N, 140.2679°E). The 15 primer pairs were also tested in 13 samples of *T. sutchuenensis* and four of *T. koraeinsis* (Additional file 1: Table S1). Genetic analyses were undertaken in GenAlEx 6.5 [9] and Genepop 4.2 [11]. In addition, a similarity search of the contigs containing the 15 loci was conducted by the BLASTX algorithm [1] against the National Center for Biotechnology Information (NCBI) non-redundant protein sequences (nr) database.

The multi locus probability of identity (PID) for the 15 markers, that is, the probability that two individuals drawn at random from a population will have the same genotype [14], was calculated in Gimlet version 1.3.3 [12] using all 97 samples from the three population of *T. standishii*. Three PID estimates outlined by Waits et al. [14] were estimated: biased PID, which assumes individuals mate randomly; unbiased PID, which corrects for sampling a small number of individuals and, sibs PID, which assumes the population is composed of siblings.

### Results

In *T. standishii*, the 15 loci (Table 1) displayed 3 to 21 alleles over the three populations with an average of 9.2 alleles per locus (Table 2). Overall observed heterozygosity varied between 0.33 and 0.75 (average = 0.54) while expected heterozygosity values were generally higher (0.37–0.91 with an average of 0.62). At the population level, the number of alleles observed per locus varied from 2 to 15 (average = 5.43) with six loci showing more than four alleles in each of the three populations. No significant deviations from Hardy–Weinberg equilibrium expectations were detected for any loci except for Kurobe_4219 in the Atebi Daira and Mt Yamizo populations (P ≤ 0.0004). Additionally, allele frequencies appeared independent among loci with no significant linkage disequilibrium detected after Bonferroni correction.

**Table 1:** Characteristics of the 15 microsatellite markers developed for *Thuja standishii*

Locus	Primer (5′-3′)	Repeat motif	Allele size range (bp)	BlastX top hit description	E-value	Genbank accession
Kurobe_18480	F: TCAGGACCCCAACAAATAAA R: GATGCTGGAGATGGTATTTCG	(AT)9	135–189	Unknown [*Picea sitchensis*] ABR17033.1	3E−95	MN170833
Kurobe_2969	F: ACACAATCTTGCCCAGAAGC R: ACACAATCTTGCCCAGAAGC	(TA)8	269–291	WRI2 [*Larix gmelinii* var. *olgensis* x *L. kaempferi*] AJP75043.1	0	MN170828
Kurobe_23700	F: CCGACGCCTCTACATTCATT R: ATGGAAAAGAGGCAGAGCAA	(TCT)10	250–268	Hypothetical protein MARPO_0043s0052 [*Marchantia polymorpha*] PTQ39804.1	2E−39	MN170835
Kurobe_44557	F: GATCCCTTTTGATGGGTTCA R: TTATCCCCAACTTCAGCGAG	(TC)11	313–349	–	–	MN170841
Kurobe_15129	F: TTAACATCCACCAATGGCAA R: CAAATGCATGGAATGTGGTC	(TC)12	205–233	Unknown [*Picea sitchensis*] ABK22564.1	9E−66	MN170831
Kurobe_16758	F: TGCAACGTCATAACCACGAT R: CACCCTTCTAGAGCTGGTCG	(CT)8	272–283	CYP866B7 [*Taxus chinensis*] ATG30002.1	0	MN170832
Kurobe_6943	F: CTACGTCTTAGCCTCCACGC R: ATGCCATACTGGGGTCTGAG	(CT)11	159–169	Early nodulin-like protein 2 [*Amborella trichopoda*] XP_011628802.2	4E−37	MN170830
Kurobe_23263	F: CCAGGCCCTAATTTCCACTC R: CCATGGCACATGGATACAGA	(AG)10	290–314	–	–	MN170834
Kurobe_51603	F: CAAAGCAACAGAGCCAACAA R: AAATAGGGTGCTGCAGGTTG	(TG)13	162–201	–	–	MN170842
Kurobe_31302	F: TCCCAAGTCTGGGGCAGT R: GTCACAGTCGGGAGCAATTT	(GT)8	264–274	–	–	MN170836
Kurobe_38308	F: TTTGCTGAAACCCAGGAATC R: CCCCATATGGAAGAAGATCC	(TG)11	272–297	–	–	MN170837
Kurobe_41636	F: TTGAACTTTGCAGTGGGAAA R: GAACCAGACAGGAATCGGAA	(CA)8	157–165	–	–	MN170839
Kurobe_42400	F: TTCACACAAGGGCCTACTCC R: GCCGACAGGTCTCTTAGCAC	(TC)9	254–272	–	–	MN170840
Kurobe_40825	F: TTATCCCATGAGCGTGCTTT R: TAGGTGCGGTGACTATGCAG	(CT)8	232–276	–	–	MN170838
Kurobe_4219	F: ACGCATCGGACAGTCGTATT R: CCGTGCAGAACCAAAGAAAT	(TC)8	276–306	MACPF domain-containing protein At4g24290 [*Morus notabilis*] XP_010087924.1	0	MN170829

– denotes that no BlastX hits were found

**Table 2:** Genetic diversity of the 15 polymorphic nuclear microsatellites assessed across the three populations of *Thuja standishii*

Locus	Atebi Daira (32)			Mt Torigata (31)			Mt Yamizo (34)			All (97)		
	*Na*	*Ho*	*He*	*Na*	*Ho*	*He*	*Na*	*Ho*	*He*	*Na*	*Ho*	*He*
Kurobe_18480	13	0.75	0.77	11	0.81	0.82	8	0.38	0.43	21	0.64	0.77
Kurobe_2969	6	0.53	0.45	5	0.52	0.60	7	0.85	0.77	9	0.64	0.75
Kurobe_23700	5	0.78	0.77	5	0.42	0.38	6	0.59	0.57	7	0.60	0.66
Kurobe_44557	14	0.68	0.89	9	0.68	0.73	15	0.88	0.88	21	0.75	0.91
Kurobe_15129	7	0.66	0.71	2	0.03	0.03	5	0.68	0.69	8	0.46	0.63
Kurobe_16758	4	0.50	0.51	3	0.29	0.26	4	0.85	0.73	7	0.56	0.60
Kurobe_6943	3	0.28	0.30	3	0.65	0.51	4	0.35	0.33	7	0.42	0.50
Kurobe_23263	2	0.41	0.46	3	0.48	0.44	3	0.38	0.51	4	0.42	0.52
Kurobe_51603	7	0.75	0.75	3	0.55	0.54	8	0.82	0.70	9	0.71	0.72
Kurobe_31302	3	0.63	0.55	3	0.52	0.51	2	0.12	0.11	3	0.41	0.42
Kurobe_38308	6	0.59	0.66	3	0.55	0.45	5	0.50	0.58	9	0.55	0.61
Kurobe_41636	2	0.41	0.36	2	0.39	0.46	3	0.21	0.21	4	0.33	0.37
Kurobe_42400	7	0.50	0.48	5	0.58	0.53	6	0.38	0.36	9	0.48	0.46
Kurobe_40825	7	0.50	0.63	6	0.71	0.71	5	0.68	0.58	11	0.63	0.73
Kurobe_4219	4	0.28	0.51	6	0.61	0.57	6	0.62	0.70	9	0.51	0.61
Average	6.00	0.55	0.59	4.60	0.52	0.50	5.80	0.55	0.54	9.20	0.54	0.62

*Na* the number of alleles, *H*_*o*_ observed heterozygosity, *H*_*e*_ expected heterozygosity

Multi-locus probability of identity values were below the threshold value of 0.01 considered by Waits et al. [14] to be required to reliably distinguish between individual genotypes, even under the sibs PID (Additional file 1: Table S2). This indicates that our markers will be effective for both identifying individuals derived from clonal reproduction and sexually derived individuals in populations even where inbreeding is prevalent.

All 15 loci amplified in *T. sutchuenensis* (two loci being monomorphic) with 1 to 11 alleles per locus (average = 4.33) while average observed and expected heterozygosity were 0.43 and 0.48, respectively (Table 3). On the other hand, only 13 loci amplified in *T. koraiensis* with three loci being monomorphic. In this species, 1 to 5 alleles per locus (average = 2.15) were found with average observed and expected heterozygosity of 0.44 and 0.31, respectively (Table 3).

**Table 3:** Genetic diversity of the 15 microsatellite loci in *T. sutchuenensis* and *T. koraiensis*

Locus	*T. sutchuenensis* (13)			*T. koraiensis* (4)		
	*Na*	*Ho*	*He*	*Na*	*Ho*	*He*
Kurobe_18480	7	0.46	0.80	1	0.00	0.00
Kurobe_2969	7	0.61	0.79	2	1.00	0.50
Kurobe_23700	3	0.15	0.14	2	0.25	0.22
Kurobe_44557	11	0.61	0.89	3	0.50	0.41
Kurobe_15129	6	0.69	0.76	2	0.25	0.22
Kurobe_16758	4	0.69	0.56	–	–	–
Kurobe_6943	1	0.00	0.00	–	–	–
Kurobe_23263	3	0.38	0.33	2	0.50	0.50
Kurobe_51603	4	0.38	0.38	5	1.00	0.75
Kurobe_31302	1	0.00	0.00	2	0.75	0.47
Kurobe_38308	6	0.61	0.69	2	0.25	0.22
Kurobe_41636	2	0.46	0.43	2	0.25	0.22
Kurobe_42400	2	0.31	0.26	1	0.00	0.00
Kurobe_40825	6	0.69	0.80	1	0.00	0.00
Kurobe_4219	2	0.38	0.39	3	1.00	0.59
Average	4.33	0.43	0.48	2.15	0.44	0.31

– denotes that the loci did not amplify

### Discussion

The development of EST microsatellites for *Thuja standishii* will enable new genetic research into this important Japanese endemic conifer including studies of range-wide level genetic diversity and gene flow and also stand-level processes such as inbreeding and clonality. The development of molecular markers may help to foster research into this species, which because of its wide ecological range, from warm temperate forests to near the alpine zone, is an ideal species to investigate ecological and genetic processes under strongly contrasting climates.

The transferability of the 15 loci was consistent with the phylogenetic relationships of the East Asian *Thuja* [7, 10]. Thus, all 15 loci successfully amplified in the sister species of *T. standishii*, *T. sutchuenensis*, and displayed considerable allelic diversity with up to 11 alleles per locus. These loci, therefore, may be particularly applicable for use in genetic studies of this geographically restricted endangered species [16]. In contrast, two of the fifteen loci did not amplify in the more distantly related *T. koraiensis* and the number of alleles per locus (ranging from 1 to 5 alleles) was low although this low allelic diversity is likely to also be due to the low number of samples tested.

## Limitations

- The number of published microsatellite markers may be too low for optimal performance of some genetic analyses.
- These microsatellite loci have not been tested in the two North American species, *T. plicata* and *T. occidentalis*.
- We did not afford much time optimizing loci, therefore some polymorphic loci that may have worked with further effort may have been excluded.

## Supplementary information

**Additional file 1: Table S1.** Sampling information for *T. sutchuenensis* and *T. koraiensis*. Apart from two samples from Halla Arboretum on Jeju Island, all samples were derived from natural populations. **Table S2.** The probability of identity (PID) values for each of the 15 polymorphic loci per locus and the multi-locus values.

## Data Availability

The 38,076,160 paired-end reads are deposited in the NCBI BioProject Database, Accession number: PRJNA554973. The genotype data for every individual are available on demand to the corresponding author.

## Abbreviations

bp: base pair
EST: expressed sequence tag
RNA: ribonucleic acid
