# Supplementary material for: Development of microsatellite markers for the Japanese endemic conifer Thuja standishii and transfer to other East Asian species
Source: BMC Res Notes. 2019 Oct 25;12:694. doi: 10.1186/s13104-019-4716-z (PMC6814961; doi:10.1186/s13104-019-4716-z)
Supplement: Supplementary file 1 — Additional file 1: Table S1. Sampling information for T. sutchuenensis and T. koraiensis. Apart from two samples from Halla Arboretum on Jeju Island, all samples were derived from natural populations. Table S2. The probability of identity (PID) values for each of the 15 polymorphic loci per locus and the multi-locus values. [file 13104_2019_4716_MOESM1_ESM.docx]

**Table S1** Sampling information for *T. sutchuenensis* and *T. koraiensis*. Apart from two samples from Halla Arboretum on Jeju Island, all samples were derived from natural populations.

| Species | *N* | Population | Location | | Elevation (m) | | Latitude | | Longitude | |  |
| --- | --- | --- | --- | --- | --- | --- | --- | --- | --- | --- | --- |
| *T. sutchuenensis* | 3 | Xuebaoshan 1 | | Manyue, Kaizhou, Chongqing | | 2152 | | 31.6063 | | 108.666 | |
| *T. sutchuenensis* | 2 | Xuebaoshan 2 | | Guanmian, Kaizhou, Chongqing | | 2156 | | 31.6166 | | 31.6166 | |
| *T. sutchuenensis* | 5 | Daba Mountain | | Xianyi, Chengkou, Chongqing | | 1639 | | 31.649 | | 31.649 | |
| *T. sutchuenensis* | 3 | Xuanhan | | Xuanhan, Sichuan | | 1601 | | 31.6354 | | 31.6354 | |
| *T. koraiensis* | 2 | Cultivated in Halla Arboretum | | Jeju Island | | - | | - | | - | |
| *T. koraiensis* | 1 | Mt.Seorak | | Gangwon Province | | 1370 | | 37.7308 | | 128.462333 | |
| *T. koraiensis* | 1 | Mt.Gyebang | | Gangwon Province | | 1367 | | 38.124028 | | 128.393222 | |

**Table S2** The probability of identity (PID) values for each of the 15 polymorphic loci per locus and the multi-locus values.

|  | **Probability of identity** | | |
| --- | --- | --- | --- |
| **Locus** | **Biased** | **Unbiased** | **Sibs** |
| Kurobe_18480 | 0.066 | 0.059 | 0.379 |
| Kurobe_2969 | 0.100 | 0.095 | 0.401 |
| Kurobe_23700 | 0.149 | 0.141 | 0.455 |
| Kurobe_44557 | 0.014 | 0.012 | 0.298 |
| Kurobe_15129 | 0.195 | 0.188 | 0.486 |
| Kurobe_16758 | 0.200 | 0.191 | 0.501 |
| Kurobe_6943 | 0.295 | 0.285 | 0.576 |
| Kurobe_23263 | 0.347 | 0.344 | 0.578 |
| Kurobe_51603 | 0.116 | 0.109 | 0.421 |
| Kurobe_31302 | 0.380 | 0.371 | 0.634 |
| Kurobe_38308 | 0.230 | 0.224 | 0.505 |
| Kurobe_41636 | 0.437 | 0.429 | 0.673 |
| Kurobe_42400 | 0.310 | 0.298 | 0.597 |
| Kurobe_40825 | 0.108 | 0.101 | 0.414 |
| Kurobe_4219 | 0.201 | 0.193 | 0.495 |
| Multi-locus value | 0.000000000002 | 0.000000000001 | 0.000018523250 |
